# A new insight into the impact of systemic lupus erythematosus on oocyte and embryo development as well as female fertility

**DOI:** 10.3389/fimmu.2023.1132045

**Published:** 2023-03-23

**Authors:** Ruolin Mao, Xiangfei Wang, Rui Long, Meng Wang, Lei Jin, Lixia Zhu

**Affiliations:** Reproductive Medicine Center, Tongji Hospital, Tongji Medical College, Huazhong University of Science and Technology, Wuhan, China

**Keywords:** systemic lupus erythematosus (SLE), oocyte and embryonic development, ovarian reserve, cumulative live birth rate, *in vitro* fertilization

## Abstract

**Background:**

Systemic lupus erythematosus (SLE) is often associated with adverse reproductive outcomes. But it’s currently unclear regarding the role of SLE in oocyte and embryonic development. Also, it’s controversial whether SLE has an adverse effect on fertility. There is a lack of comprehensive understanding and assessment of fertility in patients with SLE.

**Objective:**

This study was aim to investigate oocyte and embryonic development as well as ovarian reserve, and clinical outcomes in SLE patients during *in vitro* fertilization (IVF) treatment. By combining data on embryonic and gamete development in SLE patients, we hope to provide new insights into a comprehensive assessment of fertility in SLE patients.

**Methods:**

In this study, we collected data from 34 SLE patients who were previously diagnosed and in remission for a total of 44 IVF cycles and matched 102 infertile women with a total of 148 IVF cycles by Propensity Score Matching (PSM) of 1:3 ratio. We then evaluated baseline characteristics, ovarian reserve, IVF laboratory outcomes, and clinical outcomes between the two groups.

**Results:**

After PSM matching, baseline characteristics including age, infertility types, and duration, as well as infertility causes overall coincided between the two groups. Anti-müllerian hormone (AMH) was significantly lower in the SLE group *vs* comparison (1.9 *vs*. 3.3 ng/mL, *P*=0.001). The SLE group performed a significant reduction in available embryo rate (76.6% *vs*. 86.0%, *P*=0.001), good-quality blastocyst formation rate (35.1% *vs*. 47.0%, *P*=0.003), and blastocyst formation rate (51.0% *vs*. 67.7%, *P*=0.001) compared to the comparison. As for clinical outcomes, the implantation rate in the SLE group was notably lower (37.9% *vs*. 54.9%, *P*=0.022). The CLBR following every embryo-transfer procedure was distinctly lower (41.2% *vs* 64.7%, *P*=0.016) in the SLE group *vs* comparison. Also, the conservative and optimal CLBRs following every complete cycle procedure were significantly reduced in the SLE group *vs* the comparison (*P*=0.001, both).

**Conclusion:**

Patients with SLE present worse outcomes in oocyte and embryonic development, thus yielding compromised female fertility and clinical pregnancy. Individualized fertility assessment and early fertility guidance are necessary for these special groups.

## Introduction

Immunological infertility is well-known to be an important cause of female infertility and has received increasing attention in recent years (1). There is much evidence of a strong link between autoimmune diseases and reproductive failure (2). Systemic lupus erythematosus (SLE) is characterized by abnormal activation of the immune system, leading to a range of clinical manifestations from mild joint pain to severe life-threatening organ damage (3). In particular, pregnancy may exacerbate this process, resulting in serious reproductive consequences. Commonly, it is thought that SLE is associated with poor fertility outcomes, but most of the conclusions may be based on the combined impact of the original disease and the use of cytotoxic drugs (4–6) as well as the older age at which treatment results in delayed fertility. Previous studies had demonstrated the worse maternal and fetal outcomes of patients with SLE (7, 8). Generally speaking, SLE increases the risk of pregnancy complications and adverse neonatal outcomes such as eclampsia, hypertension, nephritis, miscarriage, and preterm delivery (9–11).

Nevertheless, the role of SLE disease in oocyte and embryonic development remains unclear. Also, it’s currently controversial whether SLE has a deteriorating effect on female fertility. For one thing, there are limited data on the oocyte and embryonic development in women with SLE as direct observations on gamete and embryonic development are not available due to physiological limitations. But the development of *in vitro* fertilization and embryo transfer (IVF-ET) technology has made it possible to visualize the development process of gametes and embryos *in vitro*. For another, previous studies have observed that women diagnosed with SLE could perform a decreasing ovarian reserve even if the effects of cytotoxic drugs are excluded (12–14). Whereas other studies came to the opposite conclusion. Clowse et al. noted that the household size of females with SLE did not appear to decrease (15). And another longitudinal study suggested that ovarian reserve did not differ between SLE patients and healthy women (16). Thus, research on this component is also needed to clarify.

The current study systematically evaluated the oocyte and embryonic development as well as ovarian reserve, clinical outcomes between SLE patients and the comparisons. With the first-hand evidence of oocyte and embryonic development results, this study could help us understand the fertility of SLE patients from multiple aspects, and offer evidence to assist embryologists and clinicians in dealing with SLE patients counseling and providing fertility instruction more consciously and rationally.

## Material and methods

### Study design and population

It was a single-center retrospective cohort study. All women undergoing IVF/intracytoplasmic sperm injection (IVF/ICSI) cycles from January 2013 to September 2022 in the Reproductive Medicine Center of Tongji Hospital, Tongji Medical College, Huazhong University of Science and Technology in China were reviewed.

**Figure 1:**
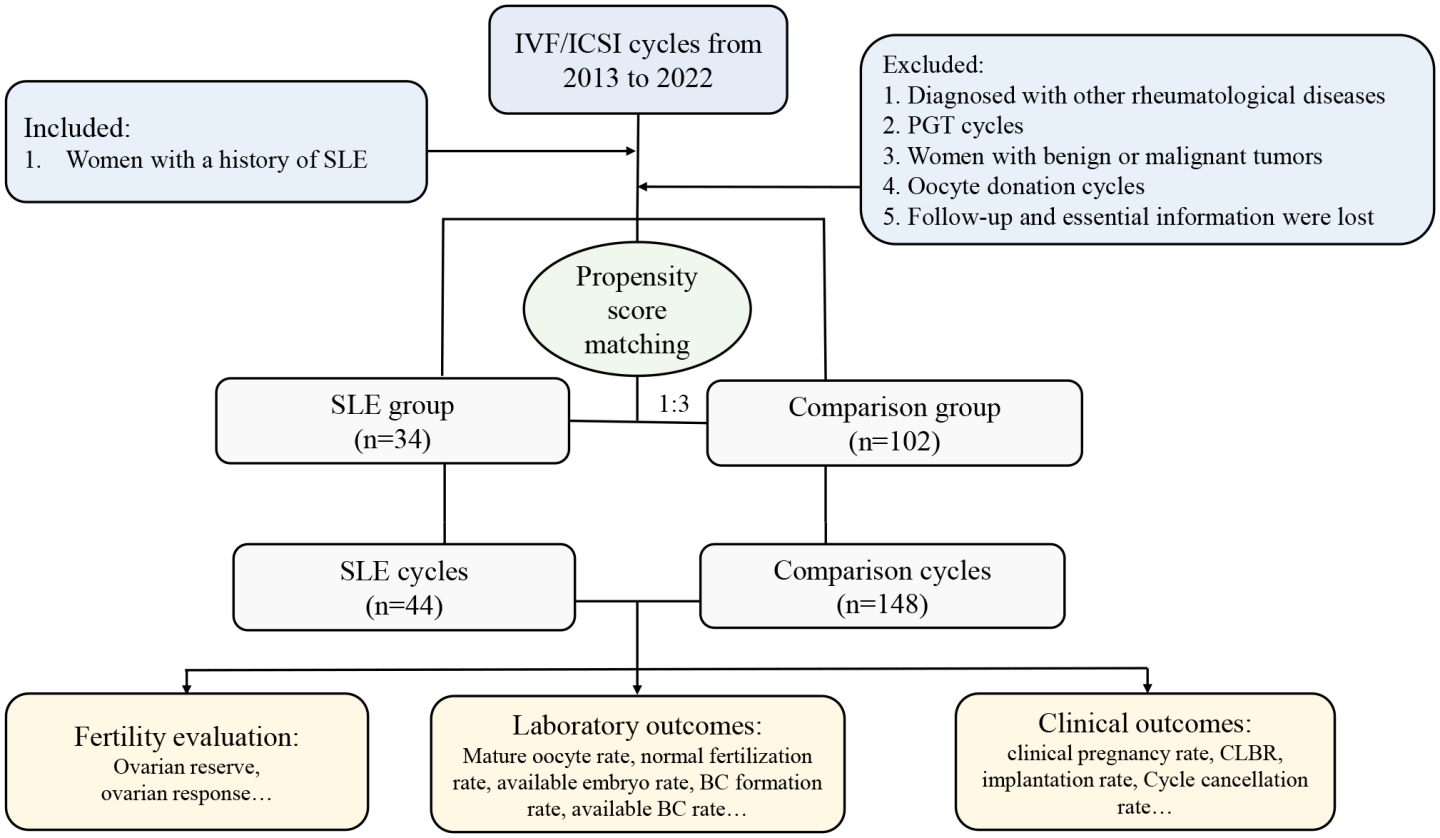
Flow chart of the present study. IVF, *in vitro* fertilization, ICSI, intracytoplasmic sperm injection; SLE, systemic lupus erythematosus; PGT, preimplantation genetic testing; CLBR, cumulative live birth rate; BC, blastocyst.

Thirty-four women with a history of rheumatologically confirmed SLE were included in the SLE group. All included SLE patients were clinically diagnosed as SLE in the department of rheumatology before they were treated with ART. The classification criteria for SLE patients were based on the 2012 SLICC criteria (17), in which patients met at least 4 classification criteria, including at least one clinical criterion and one immunological criterion, to be diagnosed with SLE. The included SLE patients have been evaluated by rheumatologists and reproductive clinicians before performing IVF techniques to ensure they were in remission (clinical remission or complete remission) for at least 6 months. Patients included in this study were considered to be in remission if they met the following conditions: (1) SLE Disease Activity Index (SLEDAI) = 0, (2) physician global assessment (PGA)< 0.5, (3) glucocorticoid dose ≤ 5 mg/d (prednisone for example) and no significant organ damage, and (4) SLE stable for ≥ 6 months. The criteria for exclusion were as follows: (1) diagnosed with other rheumatological diseases; (2) women with benign or malignant tumors; (3) preimplantation genetic testing (PGT) cycles; (4) oocyte donation cycles; (5) follow-up and essential information lost. All had been evaluated by clinicians to ensure that they were in the rest phase of the disease before IVF/ICSI cycles. Considering the complexity of etiology in SLE patients undergoing assisted reproductive technique (ART), to eliminate the potential confounders in the SLE patients and comparisons, a propensity score matching (PSM) of 1:3 ratio was performed to balance the distribution of sample and clinical characteristics. Specifically, when SLE patients with diseases of the potentially affected ovarian reserve including endometriosis, polycystic ovarian syndrome (PCOS), and ovarian surgery, the comparison was also correspondingly matched. Details about the patient’s enrollment and comparison were shown in **Figure 1**.

### Ethical approval

The original study was approved by the Ethical Committee of Tongji Hospital, Tongji Medicine College, Huazhong University of Science and Technology (TJ-IRB20211280). All the related data were extracted from the electronic medical record. Each of the patients had given written informed consent at the time of treatment for the future use of their clinical data.

### Ovarian stimulation protocol, oocyte retrieval, and embryo transfer

Ovarian stimulation protocols were processed as previously described (18, 19). Briefly, several protocols including the gonadotropin-releasing hormone (GnRH) agonist protocol, the GnRH antagonist protocols, and other protocols such as the mild stimulation and luteal phase stimulation protocols were used. The dosage and duration period of recombinant follicle-stimulating hormone (FSH) were adjusted according to the individual ovarian responses. The follicle diameter was monitored by transvaginal ultrasound. When the diameter of two or three dominant follicles exceeded 18 mm, intramuscular injection of recombinant human chorionic gonadotropin (HCG) was performed as the trigger, and later, oocytes were retrieved by guided transvaginal ultrasound 36-38h after HCG administration. Embryos may be transferred on Day 3 as appropriate after oocyte retrieval. The remaining available embryos could either be frozen on Day 3 or further cultured to Day 5 or Day 6 for cryopreservation. Cryopreserved embryos were transferred after priming the uterus with estrogen.

### Data collection

The main outcomes evaluated in the present study were demographic characteristics, ovarian reserve, gamete/embryo developmental information *in vitro*, and clinical pregnancy outcomes. For demographic characteristics, the age at cycle start, body mass index (BMI), infertility type, infertility duration, and causes of infertility were collected. Indicates of ovarian reserve included basal serum FSH level, anti-müllerian hormone (AMH) level, and antral follicle count (AFC). IVF/ICSI cycle information extracted included the amount of gonadotropin used, total days of ovarian stimulation, estradiol (E2) level, number of large follicles on human chorionic gonadotropin (HCG) trigger day, laboratory outcomes (number of oocytes retrieved, matured, fertilized, available embryos, blastocysts, good-quality embryo, embryos transferred) as well as clinical outcomes (the number of implantations, miscarriages, pregnancies, and cumulative pregnancies as well as cumulative live births).

### Criteria of assessment

Women with poor ovarian response (POR) were classified with at least two of the three following features: advanced maternal age (≥40 years) or any other risk factor for POR; exhibiting POR in a previous cycle (≤3 oocytes with a conventional ovarian stimulation protocol); and an abnormal ovarian reserve test (AFC <5–7 or AMH <0.5–1.1 ng/ml), according to the Bologna criteria (20). Women diagnosed with DOR should exist at least 2 of the following features: advanced maternal age (≥40 years); basal FSH≥12mIU/mL; AFCs≤ 5-7; AMH ≤ 1.1 ng/ml. The normal fertilization rate was the zygotes of two pronuclei (2PN) numbers divided by the number of yield oocytes; the 2PN cleavage rate was defined as the number of 2PN cleaved embryos divided by the number of 2PN zygotes; the available embryo rate referred to the ratio of the number of embryos available for transfer, cryopreservation, and extended culture divided by the number of normally-fertilized and cleaved embryos plus the late-cleaved embryos; the blastocyst formation rate was the number of blastocysts divided by the number of day 3 embryos for extended culture; the good-quality blastocyst formation rate was the blastocysts available for cryopreservation divided by the number of day 3 embryos for extended culture. The implantation rate was the ratio of the number of gestational sacs divided by the number of embryos transferred. Cycle cancellation without available oocytes was defined as one cycle receiving no available oocytes after the ovarian stimulation. Clinical pregnancy was confirmed if an intrauterine fetal heartbeat could be observed by transvaginal ultrasound. Live birth was defined as the birth of at least one live child after 28 weeks of gestation. Deliveries of multiple pregnancies were counted as one live birth. For the first-cycle cumulative live birth rate (CLBR), live birth rates (LBR) were calculated following every embryo transfer procedure during the first complete cycle. Two different types of CLBRs were calculated for several cycles. The optimal CLBR assumed that women who discontinued ART treatment would have had the same chance of having a live birth with continued ART as those who did continue, in contrast to the conservative CLBR, which was calculated based on the assumption that women who discontinued ART treatment would not have achieved a live birth if they had continued (21). Women were deemed to discontinue ART treatment if they failed to have a treatment-dependent live birth and did not return for any more ART cycles until September 30, 2022.

### Statistical analysis

Data were analyzed and presented using Statistical Package for Social Sciences software (SPSS, version 22.0, IBM, the United States) and R (version 4.1.3). Kruskal-Wallis nonparametric method was performed in the continuous data and expressed as median (interquartile range IQR), or with a student t-test if variables were normally distributed. Categorical data were presented as the number of cases and frequency (percentage), and a Chi-Square test was to assess the group differences.

The following baseline characteristics were matched by propensity score matching: age (years), body mass index (BMI, kg/m²), infertility type (primary or secondary), infertility period (years), infertility causes (male, female, or combined), previous existing ovarian related diseases such as endometriosis, PCOS and ovarian surgery. The matching algorithm was the nearest neighbor random matching without replacement, and the match ratio was 1:3 with a caliper value of 0.1. The conservative CLBR estimate was calculated as the number of live births up to and including a specific treatment cycle, divided by the number of women who started their first ART cycle during the study period. The optimal estimate of CLBR was calculated by the Kaplan-Meier method upon inclusion of all treatment cycles in the analysis. Log-rank test and Kaplan-Meier curves with live birth considered as an event were used to illustrate differences between groups (18). Wald P-values were two-sided; *P*<0.05 was considered to be significant.

## Results

### Baseline characteristics

**Figure 2:**
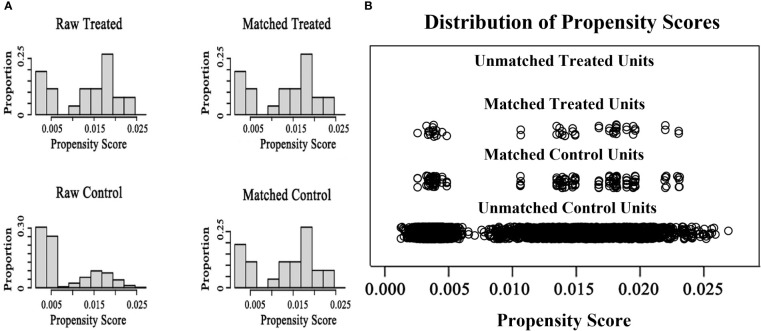
The distributions of baseline characteristics and propensity scores before and after matching **(A)** the proportion distributions of baseline characteristics in two groups before and after matching **(B)** The distributions of propensity scores in two groups before and after matching.

A total of 34 women diagnosed with SLE, involving 44 IVF/ICSI cycles, were identified and enrolled in the SLE group. All 34 patients (100%) were assessed to be in clinical remission. In addition, of the 34 patients included in the SLE group, thirty-two patients (94.1%) were treated with prednisone at a maintenance dose of 5 mg/d and hydroxychloroquine at 200mg/d during IVF procedures, two patients (5.9%) did not use any medications; Five patients (14.7%) had previously undergone pulse cyclophosphamide therapy and only one (2.9%) had a successful live birth. And twenty-three patients (67.6%) had prior lupus nephritis and most of the clinical manifestations were mild, mainly as asymptomatic hematuria and proteinuria. Women matched by PSM according to a 1:3 match ratio were included in the comparison group (matching criteria including age, BMI, type and duration of infertility, and infertility causes), resulting in 102 patients with 148 IVF/ICSI cycles (**Figure 1**). The median age of women at the start of ART was 32 years, and the number of primary and secondary types was similar. Other baseline characteristics, including BMI, type of infertility, duration, and cause of infertility, were similar between the two groups (**Table 1**). The distributions of baseline characteristics and propensity scores were presented through visualized graphics. The proportions of baseline characteristics after matching were almost identical, and the distribution of propensity scores almost coincided between the two groups after matching, confirming the validity of PSM matching (**Figure 2**).

**Table 1:** Demographic characteristics of the SLE and comparison groups.

Characteristics	SLE patients	Comparison	P-value
**Number of patients, n**	34	102	
**Number of ART cycles, n**	44	148	
**Female age at cycle start (y)**	32.0(30.0-35.0)	32.0 (30.0-35.0)	0.824
**BMI (kg/m^2^)**	21.5(20.0-24.5)	21.8 (19.7-24.1)	0.998
**Infertility duration (y)**	2(1.0-4.0)	2 (1.0-5.0)	0.924
**Infertility type, n (%)**			1.000
** Primary**	16(47.1%)	48(47.1%)	
** Secondary**	18(52.9%)	54(52.9%)	
**Infertility cause, n (%)**			0.752
** Female factors only**	24(70.6)	70(68.6%)	
** Male factors only**	0	2(2.0%)	
** Combined male and female factors**	7(20.6%)	22(21.6%)	
** Unexplained**	3(8.8%)	8(7.8%)	

Values are median (IQR) for continuous variables and n (%) for categorical variables. SLE, systemic lupus erythematosus; ART, assisted reproductive technology; BMI, body mass index.

### Ovarian reserve and response

According to the results, the SLE group had a significantly lower basal AMH level (1.9 *vs*. 3.3 ng/mL, *P*=0.001), than the comparison (**Table 2**). And the AFC was slightly lower (7.0 *vs*. 9.0, *P*=0.056) in the SLE group *vs* the comparison group. The incidence of DOR in the SLE group was significantly higher than that in the comparison group (42.2% *vs* 10.8%, *P*=0.001). As for the ovarian response, Patients in the SLE group underwent fewer days and used a less total dose of gonadotropins during controlled ovarian hyperstimulation. The rest of the results were generally consistent between the two groups. Based on the Bologna criteria, more women with a history of SLE were diagnosed as POR than those without SLE (26.5% *vs*. 14.7%), even with no significant difference (**Table 2**).

**Table 2:** Ovarian reserve and response to stimulation in the SLE and comparison groups.

Reproductive results	SLE patients	Comparison	P-value
Ovarian reserve			
** Day3 FSH**	7.7 (6.3-10.2)	7.5 (6.5-8.8)	0.905
** Day3 AFC**	7.0 (5.0-13.5)	9.0 (6.0-15.8)	0.056
** AMH (ng/mL)**	1.9 (0.3-4.0)	3.3 (1.3-5.4)	0.001
** Incidence of DOR**	42.2% (14/34)	10.8% (11/102)	0.001
Ovarian response			
** Total dose of gonadotropins (IU)**	2231.3(1500.0-2993.8)	2523.8 (2025.0-3328.1)	0.017
** Days of gonadotropins use (d)**	9.0(8.0-11.0)	10.0 (9.0-11.0)	0.094
** E2 on hCG trigger day (pg/mL)**	2590.5(879.5-4246.0)	2007.0(1224.3-2923.3)	0.354
** No. of large follicles on hCG day**	8 (2-12)	9 (5-12)	0.358
** Incidence of POR**	26.5%	14.7%	0.119

SLE, systemic lupus erythematosus; FSH, follicle-stimulating hormone; AFC, antral follicle count; AMH, antimüllerian hormone; E2, estradiol; hCG, human chorionic gonadotropin; POR, poor ovarian response; DOR, diminished ovarian reserve. Red bold fonts were statistically significant.

### Oocyte and embryo viability assessment

Regarding the IVF laboratory outcomes, the numbers of MII oocytes and oocytes retrieved between the two groups were similar. Hence, there were no differences in the maturation rate between the SLE group and the comparison (86.1% *vs* 84.3%, *P*=0.360). In addition, the normal fertilization rate and 2PN cleavage rate were comparable between the two groups (*P*= 0.841*, P*=0.104, respectively), while the available embryo rate was distinctly lower in the SLE group compared to the comparison (76.6% *vs*. 86.0%, *P=0.001*). It is worth noting that in the embryo extended culture, significant differences were observed in blastocyst formation rate (51.0% *vs*. 67.7%, *P*=0.001) and good-quality blastocyst formation rate (35.1% *vs*. 47.0%, *P*=0.003) between SLE group and the comparison (**Table 3**).

**Table 3:** IVF/ICSI laboratory outcomes and clinical outcomes of SLE and comparison groups.

IVF/ICSI outcomes	SLE patients	Comparison	P-value
Laboratory outcomes			
** No. of oocytes retrieved**	9 (2-15)	10 (5-15)	0.205
** No. of MII oocytes**	8 (2-12)	8 (4-13)	0.251
** Maturation rate**	86.1% (360/418)	84.3% (1311/1555)	0.360
** Normal fertilization rate**	61.0% (255/418)	61.5%(957/1555)	0.841
** 2PN Cleavage rate**	98.8%(252/255)	96.7% (925/957)	0.104
** Available embryo rate**	76.6%(246/321)	86.0%(936/1088)	0.001
** Good-quality blastocyst formation rate**	35.1% (71/202)	47.0% (362/771)	0.003
** Blastocyst formation rate**	51.0%(103/202)	67.7% (522/771)	0.001
Clinical outcomes			
** Cycle cancellation rate**	20.5% (9/44)	2.70% (4/148)	0.001
** No. of ET cycles**	41	189	
** No. of embryos transferred**	58	213	
** Average no. of embryos transferred**	1.41	1.13	0.101
** Implantation rate**	37.9%(22/58)	54.9% (117/213)	0.022
** Clinical pregnancy rate**	46.3% (19/41)	56.6% (107/189)	0.231
** Live birth rate per complete cycle**	45.2% (14/31)	52.2% (70/134)	0.477

SLE, systemic lupus erythematosus; CLBR, cumulative live birth rate; Red bold fonts were statistically significant.

### ART clinical outcomes

**Figure 3:**
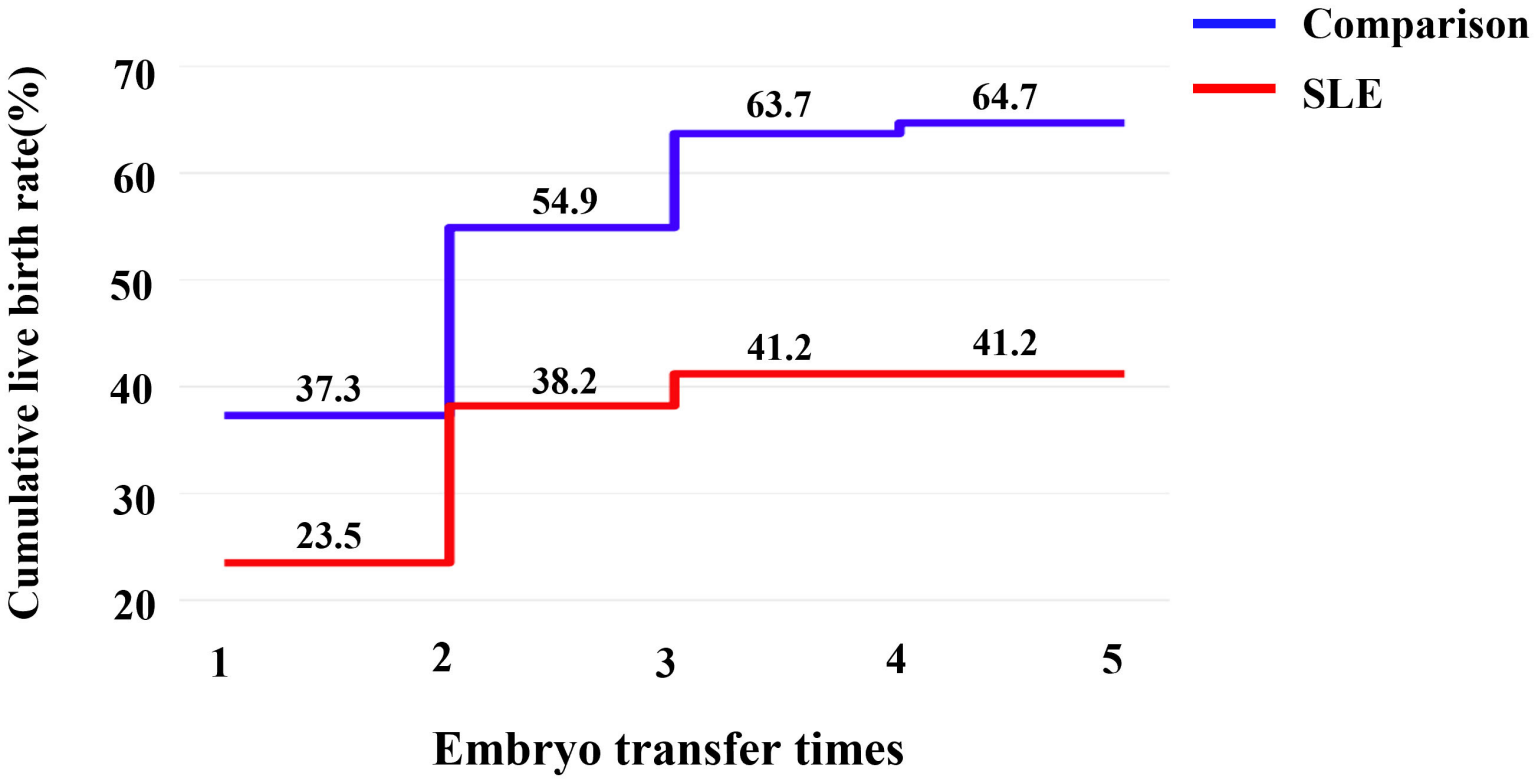
The CLBR following every embryo-transfer procedure. For each complete cycle, the LBR following every embryo-transfer procedure rose from 23.5% to 41.2% in the SLE group (red line), and from 37.3% to 64.7% in the comparison group (blue line). Significant differences were found between the two groups (*P*=0.016).

**Figure 4:**
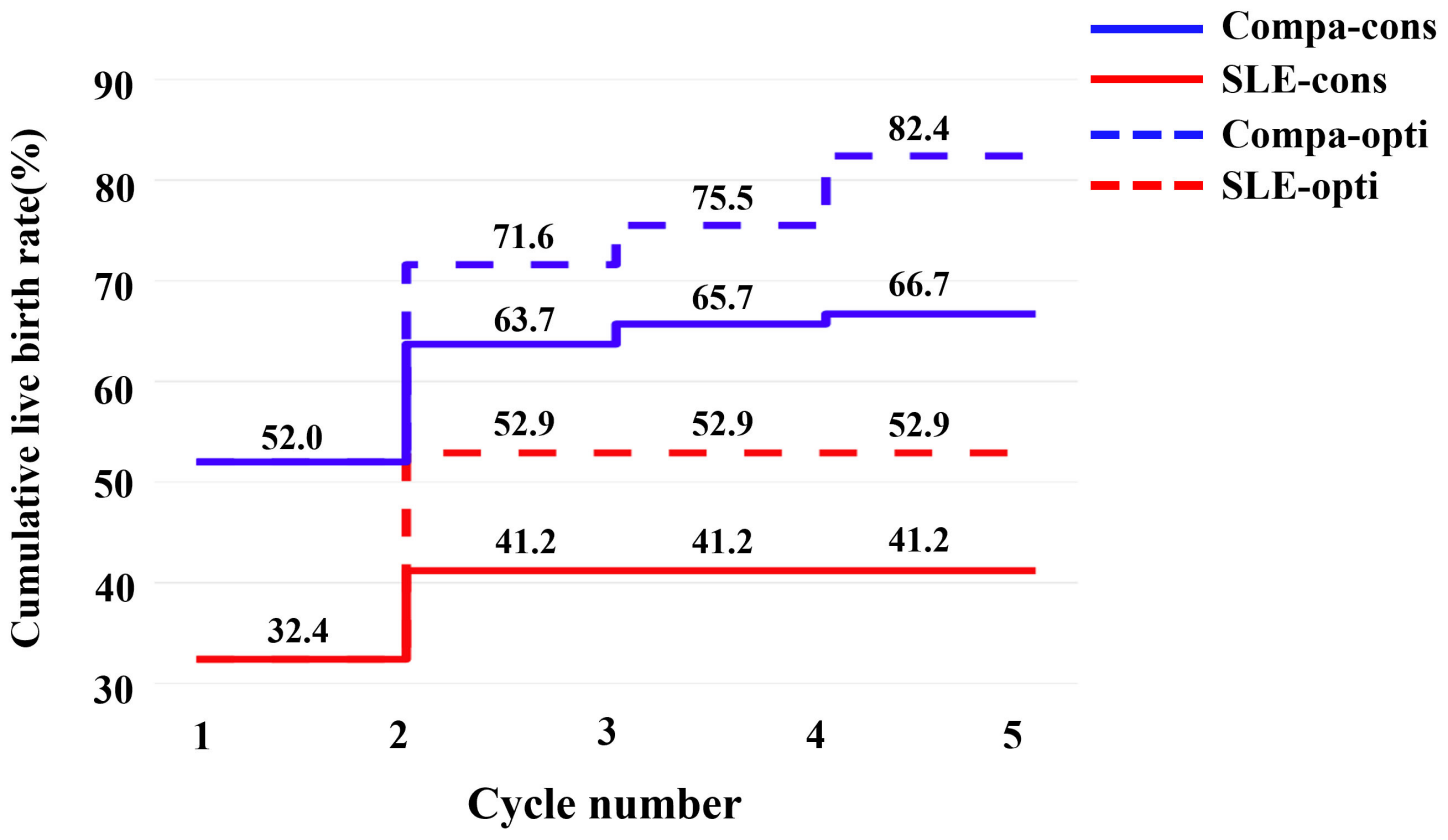
The conservative and optimal CLBRs for up to five complete cycles in two groups. The CLBR was 32.4% for the first complete cycle of the SLE group, rising to 41.2% (conservative) and 52.9% (optimal) for the second cycle. In the comparison group, the CLBR increased from 52.0% for the first cycle to 66.7% (conservative) and 82.4% (optimal) for the fifth cycle. The difference of optimal CLBRs between the two groups was significant (*P*=0.009). CLBRs did not increase from the third cycle in the SLE group.

Due to the deteriorated embryo development, 6 patients in the SLE group had to cancel a total of 9 cycles due to lack of available embryos, and the cycle cancellation rate was 20.5%, compared with 4 patients in the comparison group with a total of 4 cycles, manifesting only 2.7% of the cycle cancellation rate (*P*=0.001). For all embryo transfer cycles, the average numbers of transferred embryos were 1.41 and 1.13 in the SLE and comparison groups, respectively (*P*>0.05). And our results showed a notably lower implantation rate in the SLE group (37.9% *vs*. 54.9%, *P*=0.022), whereas the clinical pregnancy rate was slightly lower (46.3% *vs*. 56.6%, *P*= 0.231) (**Table 3**). For one complete cycle, which involves the outcomes from all fresh and following frozen/thawed embryo transfers after one ovarian stimulation, the LBR of the SLE group were slightly lower than those of the comparison group (45.2% *vs*. 52.2%, *P* =0.477) (**Table 3**). For the per complete cycle, the CLBR following every embryo-transfer procedure increased from 23.5% to 41.2% in the SLE group, and from 37.3% to 64.7% in the comparison group (*P*=0.016), as shown in **Figure 3**. The conservative and optimal CLBRs for up to fourth complete cycles were presented in **Figure 4**. In general, the CLBR of the first complete cycle in the SLE group was 32.4%, later rising to 41.2% (conservative) and 52.9% (optimal) for the fourth complete cycle, while in the comparison group, the CLBR rose from 52.0% for the first cycle to 66.7% (conservative) and 82.4% (optimal) for the fourth cycle. The difference in conservative and optimal CLBRs between the two groups was significant (*P=*0.001, both). CLBRs did not increase from the third complete cycle in the SLE group (**Figure 4**).

## Discussion

As previously mentioned, there has been more concern about the adverse effects of SLE activity and related cytotoxic drug treatment on the pregnancy process and the newborn (5), but few studies have focused on clinically diagnosed SLE women about whether the quality of their oocytes and embryonic development potential are irreversibly reduced. In the present study, after the elimination of confounding factors by PSM matching, we evaluated the impact of SLE disease on oocyte and embryo competence as well as ovarian reserve and clinical outcomes. The results of this study demonstrated that women with diagnosed SLE or a history of SLE had compromised oocyte and embryo viability and lower ovarian reserve, thus resulting in poorer IVF clinical outcomes such as a higher cycle cancellation rate, impaired embryo implantation, and reduced CLBR compared to common infertility women who underwent IVF/ICSI. We provided new insight into fertility assessment in SLE patients in terms of evident observation of oocyte and embryo development.

To begin with, the oocyte and embryo viability in SLE patients has been adversely affected. The rates of the available embryo, blastocyst formation, and good-quality blastocyst were quite lower in SLE patients than in the comparison group. The above results suggested that SLE has exerted a detrimental effect on oocyte and embryo developmental competence. We could reasonably speculate that several reasons could contribute to the poor results. As mentioned earlier, elevated antinuclear antibody (ANA) titers are known to be the most important feature of SLE (22). Early in 1990, a study reported that murine embryos exhibited growth arrest and sustained fertilization failure when murine embryos were co-cultured with purified IgG from ANA (+) individuals (23). A work by Simerly et al. also suggested the direct harmful effects of ANA on embryo development (24). This damage may continue to impair embryo formation and available embryos, resulting in a significant reduction in high-quality embryos. Moreover, Ying et al. compared IVF laboratory outcomes in ANA (+) and ANA (-) patients, and the results indicated the rates of cleavage, fertilization, available embryos and, high-quality embryos in the ANA (+) group were significantly lower than those in the ANA (−) group (25–27). In addition, ANA (+) patients had lower implantation rates and pregnancy rates in subsequent embryo transfers (ET) (25). These series of studies concluded that the presence of ANAs interfered with the oocyte quality and embryo development, reducing the success of implantation and pregnancy. The specific mechanism by which ANA interferes with oocyte quality and embryonic development was not well understood, and previous studies generally suggested that it may be related to abnormal centromeres and damaged cytoskeleton (24, 28). Additionally, studies indicated that patients with SLE are often accompanied by mitochondrial dysfunction (29, 30), intracellular reactive oxygen species (ROS) accumulation, and endoplasmic reticulum stress (31). All the alterations may further affect subsequent oocyte development and embryo formation, resulting in poor IVF laboratory outcomes in SLE patients. Furthermore, there is a variety of different autoimmune antibodies except for ANA in SLE patients, each of which may play a special role in the mechanism of the ovarian dysfunction. For instance, anti-small nuclear RNA antibodies (anti-snRNA) may affect the recognition of misfolded RNA by binding the small RNAs, leading to unknown cell disorders (32). However, the exact mechanism remains largely unclear, and more studies are required in the future.

The deleterious effects of SLE on ovarian reserve seem to be expected. It’s well-known that SLE could cause a systemic inflammatory immune response through the accumulation of antigen-antibody complexes and activation of inflammatory factors (22), which may directly impair the ovary. Similar to our study, previous studies had shown that the AMH level of SLE patients was significantly lower than normal infertile women (4, 12), even without the extra use of cytotoxic drugs. The linear regression model also confirmed the association between SLE and low AMH in the absence of treatment with cytotoxic drugs (14). Other than that, Pasoto et al. found a significantly higher risk of menstrual disorders in women with SLE who were not treated with cytotoxic drugs. Reduced AMH and a high incidence of menstrual abnormalities suggest that autoimmune activity in SLE could impair ovarian reserve in female patients (33, 34). The underlying mechanisms may include autoimmune ovarian injury and endocrine interactions.

Autoimmune oophoritis is one of the most common forms of autoimmune ovarian injury (35). And auto-antibodies against different targets in the ovary, such as gonadotropin receptors, corpus luteum, zona pellucida, theca cells, and oocytes, are thought to be responsible for it. Ovarian inflammation and damage induced by part of autoantibodies can also lead to decreased ovarian reserve (DOR) or premature ovarian failure (POF) (36). Meanwhile, there was much evidence of immune imbalance in patients with SLE, such as regulatory T cell dysfunction (37) and abnormal production of multiple cytokines (38) which contributed to decreased ovarian reserve. In addition, a study evaluated the lymphocyte subpopulation in SLE patients. The study indicated the percentages of CD4^+^ T lymphocytes and natural killer (NK) cells in SLE patients were significantly lower than in the normal group, whereas the percentages of B cells and CD8^+^ T lymphocytes were much higher (34). Subsequent disturbances in multiple cytokines and signaling pathways in the ovary environment contribute to ovarian damage. Therefore, A state of autoimmune ovarian injury in SLE patients may play a key role in the decline of ovarian reserve. In addition, hypothalamic-pituitary-ovarian (HPO) axis disorders due to SLE also account for a proportion of impaired ovarian reserve. The chronic inflammatory state may destroy the normal functioning of the HPO axis through endocrine interactions. Studies had revealed that prolactin (PRL) was related to SLE activity, which could interfere with normal ovulation (39). Apart from this, part of the studies indicated aberrations in the frequency and amplitude of GnRH pulses and supported that autoimmune disease was strongly correlated with HPO axis disorder (40, 41). Disruption of the HPO axis inevitably affected ovarian function and oocyte development, leading to a decrease in ovarian reserve.

For the ovarian response, SLE patients had overall higher E2 levels despite lower gonadotropin dosing. A possible explanation was the interaction between SLE disease and E2. There was much evidence to support that E2 was considered to be the predisposing factor of SLE, and played a crucial role in the pathogenesis of SLE (5, 42). Additionally, studies have found that single-nucleotide polymorphisms (SNPs) showed a gender bias between different sexes (43). Parts of them could be involved in sex hormone and immune system signaling, as well as genes related to the immune system, which may also be the reason why SLE patients showed higher E2 levels. What’s more, although there was no distinct difference in maturation rate, the patients with SLE received fewer oocytes retrieved and MII oocytes. The possible causes may be the low-dose use of gonadotropins, but combined with the diminished ovarian reserve and laboratory outcomes, it is more likely that patients with SLE would have fewer oocytes retrieved and matured oocytes. In addition, previous studies demonstrated autoimmune antibodies impaired oocyte maturation (25, 27). There was a higher incidence of POR in the patients with SLE versus individuals of comparison from our results, which may also be the result regarding the effect of SLE on ovarian.

When it comes to clinical outcomes, the rates of implantation, clinical pregnancy, and cumulative live birth in the SLE group indicated in our study were unsatisfactory. There was much evidence indicating a negative impact on the embryo implantation process with SLE. SLE can not only impair the implantation rate by affecting oocyte quality and embryonic potential, but can also reduce endometrial receptivity and change the immune environment, resulting in implantation failure (44). Quite a few studies discussed that auto-antibodies could reduce implantation rates by impairing oocyte quality and embryonic development (25, 45), which were also consistent with what we mentioned earlier. What’s more, A previous study presented that altered maternal decidual immunity in SLE patients can lead to implantation impaired and failure (46), thus immunoregulation drugs could be used to assist with embryo implantation. Overall, SLE could be generally detrimental to embryo implantation. Furthermore, similar to our study, there have been previous studies demonstrating declined live birth rate in patients with SLE (11, 47). A large retrospective cohort study reported that women with SLE had generally fewer live births than the general population (48). In addition, another meta-analysis also supported that successful live birth significantly favored women without SLE (49). The conservative CLBR is pessimistic, while the optimal CLBR is perhaps overly optimistic. Based on their computational principles, the prognosis-adjusted CLBRs were more precise to the optimal than the conservative estimate (21), we have more reason to believe that the SLE group did suffer from a decreased CLBR.

Lastly, the impact of SLE activity and related renal diseases on female fertility should not be overlooked. Nusbaum et al. reported that active disease, immunosuppressive drugs, and age-related effects could lead to declined women’s fertility (5). Similarly, Martins et al. found that female fertility was statistically associated with disease length and SLE activity by performing generalized linear univariate and multivariate models (50). Although the women with SLE included in our study were only allowed to undergo IVF/ICSI after being assessed by clinicians to be in the resting phase, we still needed to pay attention to the impact of SLE activity on female fertility. Additionally, impaired renal function of SLE patients is known to decrease fertility. Twenty-three patients in our study had prior lupus nephritis and most of the clinical manifestations were mild, mainly as asymptomatic hematuria and proteinuria. But due to the small sample size, we did not make further grouping and subgroup analysis. Hence, more studies on oocyte and embryonic development in these patients are required.

## Strength and limitation

### Strength

The present study first presented a new insight into the impact of SLE on oocyte and embryo viability, while other researchers had usually focused on obstetric and fetal complications of SLE. Meanwhile, the study also took the ovarian reserve and clinical outcomes of SLE patients into consideration, which was a comprehensive summary of the effects of SLE on female fertility. In addition, the comparison group excluded possible interferences such as a history of endometriosis, PCOS, and ovarian surgery by PSM matching. Lastly, both conservative and optimistic CLBRs were calculated to demonstrate the ART outcomes that patients were most concerned about.

### Limitation

This study also has several certain limitations. For one thing, it’s a retrospective and single-center study, which is relevant to an inevitable risk of bias. For another, five patients had been treated with cytotoxic drugs, which may also potentially affect their ovarian function. Besides, information on aPL (antiphospholipid antibodies) and associated APS (antiphospholipid syndrome) was not available, which may have an impact on the ART results. Lastly, due to its special population selection, the sample size of this study is quite small.

## Conclusion

In conclusion, our study has shown that women with a clinical diagnosis of SLE have worse oocyte and embryonic development, even in remission. Also, women with SLE demonstrated worse ovarian reserve and clinical pregnancy outcomes. This study provides data support to further explore the impact of SLE and other autoimmune diseases on female fertility, and it also calls for more attention to be given to these patients by reproductive physicians and rheumatologists, as well as timely intervention for fertility impairment. Comprehensive fertility assessment and followed individualized fertility guidance are recommended for patients with a history of SLE. More large-scale and multi-center studies are required to further investigate the mechanisms of the effects of SLE on female fertility.

## Data availability statement

The original contributions presented in the study are included in the article/supplementary material. Further inquiries can be directed to the corresponding authors.

## Ethics statement

The studies involving human participants were reviewed and approved by the Ethical Committee of Tongji Hospital, Tongji Medicine College, Huazhong University of Science and Technology (TJ-IRB20211280). Written informed consent for participation was not required for this study in accordance with the national legislation and the institutional requirements.

## Author contributions

LZ provided intellectual input and supervision throughout the study. RM and XW collected the clinical data. RM, XW and RL contributed to the statistical analysis. RM, LZ, XW and MW composed the manuscript. LZ and LJ were responsible for the concept and study design. All authors contributed to the article and approved the submitted version.
